# A novel set of Austrian reference unit costs for comprehensive societal perspectives consistent with latest European costing methods for economic evaluations

**DOI:** 10.1007/s00508-022-02128-6

**Published:** 2022-12-23

**Authors:** Michael Berger, Susanne Mayer, Judit Simon

**Affiliations:** 1 Department of Health Economics, Center for Public Health, Medical University of Vienna, Kinderspitalgasse 15/1, 1090 Vienna, Austria; 2 Department of Psychiatry, University of Oxford, Warneford Hospital, OX3 7JX Oxford, UK

**Keywords:** Health and social care, Education sector, Justice sector, Employment and productivity, Informal care

## Abstract

Decision making in public health often happens against the background of scarce resources. The systematic use of economic evaluations can be a main enabler in the alignment of public health goals with budgetary constraints. However, the lack of standardized methodology in terms of costing method and perspective are a critical barrier to the implementation of economic evaluations and the international comparability of results. We present a novel set of 22 reference unit costs (RUCs) optimized for cross-sectoral economic evaluations in Austria suitable for international comparability calculated using the standardized PECUNIA RUC Template. The common framework for costing and reporting, as well as the easy availability of the RUCs will reduce the burden on researchers and policy makers in future economic evaluations. The higher quality, accuracy, transparency and availability of economic evidence for policy design will help to improve the efficiency of public health-relevant healthcare decisions and make it easier for policy makers to bring funding arrangements and decision making across multiple sectors in line with Health-in-All-Policies goals.

## Introduction

Decision making in social healthcare systems with respect to public health often intersects with the fundamental problem in economics: the scarcity of resources. Although spending on healthcare in Austria has gradually increased relative to economic activity measured by the Gross Domestic Product (GDP) [1], fiscal realities impose limits on the policy makers’ possibilities to increase healthcare budgets. Economic evaluations are hence an important tool for policy makers seeking to align healthcare policy goals with budgetary constraints. In this role, economic evaluations experience increasing interest in the context of health policy making in Austria [2], although methodological and cultural challenges continue to hamper their traction [3]. Key barriers for the wider application of economic evaluations are the substantial inconsistencies in the costing methods and reporting standards [4], as well as the perceived lack of transparency in the decision-making process [5]. The choice of method can affect the cost estimates quite substantially. Mayer et al. [6] applied 6 different costing methods to the unit cost calculation of a general practitioner (GP) visit in Austria and end up with a striking 173% difference between the lowest and highest unit cost estimates.

Moreover, policy makers know that the economic impact of healthcare interventions often goes well beyond the readily observable costs directly tied to the intervention. Yet deciding when and how to include the potential spill-over costs and benefits to and from other sectors not directly related to healthcare in economic evaluations is a challenge. Again, the lack of methodological standards is a critical obstacle, as there is no consensus in the scientific community on how and when to include spill-over costs and benefits in economic evaluations [7]. It is not unreasonable to expect a similar methodological dependence of unit cost estimates in other sectors like education, employment, or informal care. This is not a new debate in health economics. In fact, it has been around for many years [8, 9] but so far the only common ground is that the benefits of the inclusion of these spill-over effects in health economic evaluations should be put in line with the intended aim and scope of a study [10–12]. While for simple budgetary decisions (e.g., funding specific technologies from a fixed budget), direct healthcare-related costs are sufficient, the value assessment of healthcare policies, treatments and interventions require a broader societal perspective. Economic evaluations with a myopic focus on healthcare costs alone may fail to account for important spill-over costs into and from other sectors, like education [13], employment [14] or both [15], criminal justice [16], or informal care [17]. Failing to account for these spill-over costs may lead to non-optimal policy choices and resource allocation from a public health viewpoint [12], which in turn may keep healthcare policy reforms from unfolding their full potential.

The magnitude of spill-over costs can be substantial. For instance, although costs related to the productivity of the patients’ paid work alone may reflect more than half of the total costs for patients with depressive disorders, they are often neglected in relevant economic evaluations of interventions [18]. Similar spill-over costs are also relevant for persons providing informal care to patients [19]. As health economic evaluations are among the essential groundwork for evidence-informed policy decision making, sensible consideration of a societal perspective is important and has been taken up in recent cost of illness studies [20, 21]. However, the quality of intersectoral, i.e., sector overarching, health economic analyses, and the resulting empirical evidence depends critically on the compatibility of the methods used in the cost assessment in the different sectors, in particular with regard to quantifying costs. The availability of such comparable unit cost estimates for Austria, though, has been limited so far [22]. In the absence of a nationwide unit cost program, the lack of a common methodological foundation for unit costs is a critical research gap.

This article presents the novel set of 22 reference unit costs (RUCs) optimized for cross-sectoral economic evaluations in Austria that were calculated in the European programme in costing, resource use measurement and outcome valuation for use in multisectoral national and international health economic evaluations (PECUNIA) [23] project in multiple sectors: health and social care, education, (criminal) justice, employment and productivity, patient and family domains and informal care. By using the PECUNIA RUCs, researchers can widen the scope of health economic evaluations in Austria across multiple sectors, while the common framework for costing methods and reporting will reduce the burden on researchers and facilitate take up by policy makers and payers in their decision-making process. The higher quality, accuracy, transparency and availability of economic evidence for policy design will contribute to improving the efficiency of public health-relevant healthcare decisions regarding reforms, service provision, and treatment choices. This will make it easier for policy makers in Austria to bring cross-sectoral funding arrangements and decision making in line with Health-in-All-Policies strategies, which aim to introduce health considerations into policymaking in all sectors [24, 25], and to better estimate the economic impact of major shocks to the healthcare system (e.g., the COVID-19 pandemic).

### The PECUNIA project

A key aim of the PECUNIA project was to develop a set of methodologically validated and standardized costing tools, including inter alia a set of RUCs [26, 27], for researchers, policy makers and professionals to apply a comprehensive societal perspective in economic evaluations of healthcare interventions, treatments or policies while maintaining methodological coherence and cross-country comparability. For this purpose, research partners from six European countries (Austria, Germany, Hungary, the Netherlands, England/UK and Spain) cooperated in the PECUNIA project coordinated by the Department of Health Economics, Medical University of Vienna, to create a methodologically validated set of RUCs based on harmonized costing methodology. The comparable methodology across countries and sectors, the comprehensive coverage of multiple sectors from a societal perspective, as well as the unambiguous identification and definition of resource use items through the PECUNIA Care Atom concept [28] and the Description and Evaluation of Services and Directories (DESDE) coding framework adapted and extended for the PECUNIA project [29, 30] in addition to comparability with the other costing tools, have been the key focus of the PECUNIA RUCs. These factors distinguish the PECUNIA methods from other existing and established unit cost programs (e.g. in the Netherlands [31], Germany [32], and England [33]), or recent databases like the European Healthcare and Social Cost Database (EU HCCD), which also includes a set of unit costs across several countries for the healthcare, although with lower focus on methodological comparability aspects. The PECUNIA tools thus allow researchers to minimize the inaccuracies in costing estimates associated with the combination of multiple costing methods or multiple sectors, and to save time by streamlining the valuation by either providing the relevant RUC estimates directly or making the necessary tools readily available for further RUC development.

The PECUNIA project framework divided the process of developing RUCs into four consecutive stages: (i) identification of relevant resource use items, (ii) definition of resource use items, (iii) measurement, and (iv) valuation. In general, each stage built on the results of the preceding one, although especially the work in the later stages (iii) measurement and (iv) valuation overlapped and was conducted in parallel. As the focus of this article lies on the newly developed cross-sectoral RUCs, we limit the discussion to the results of the valuation stage. Further details on the other steps have been published [34, 35] or are planned to be published in the near future [30, 36]. Lastly, as the PECUNIA research teams in each country worked on developing unit costs in each sector, regular sharing of the experiences in the calculation process ensured a high degree of cross-country and cross-sectoral harmonization.

## Material and methods

The piloted and validated PECUNIA RUC Templates [37] were the basis of the RUC calculations. The RUC Templates are Microsoft Excel®-based (Microsoft Corporation, One Microsoft Way, Redmond, WA, USA) step-by-step unit cost calculation blueprints for self-completion using either a top-down micro-costing or top-down gross-costing approach [38], depending on data availability and cost objective. The PECUNIA RUC Templates work with either primary and secondary data or a combination of the two data types. Each type of resource use has a dedicated RUC Template tailored to the specific costing requirements and capture all relevant cost categories. In this respect, primary and secondary data are the first best choices when calculating RUCs. In the face of limited data availability, the PECUNIA RUC Templates are also compatible with cost proxies (e.g., tariffs). However, such proxies are potentially biased and therefore only a second best option. We only used such proxies in the calculation of the RUCs presented in this article when other possibilities were exhausted.

The 16 resource use items for RUC calculation were selected from comprehensive lists of costing-relevant healthcare, social care, educational, criminal justice-related, and productivity-related resource use items [34, 39–41] according to their cost relevance in economic evaluations. The list of resource use items was conceptualized in the identification and definition stages of the PECUNIA project and aligned with the PECUNIA resource use measurement (RUM) instrument developed in the measurement stage [42]. This approach harmonizes both the units of analysis and the units of measurement and thereby increases the comparability between sectors and countries. The RUCs developed directly in the PECUNIA project are the result of the testing of the RUC templates in practice that was feasible within the timeframe of the project, and are intended to enable the wider scientific community to develop harmonized RUCs. Hence, the current set of RUCs reflects the approach of testing the RUC templates in multiple sectors rather than focussing on many different services within only one sector. Since the official end of the PECUNIA project, there have been multiple projects using the PECUNIA templates. These ongoing collaborations using the PECUNIA tools will expand the RUCs for different domains.

The following sections provide details on the three main steps of the valuation phase: data collection, RUC calculation, and external validation.

### Data collection

In the first step of the PECUNIA RUC calculation, we collected data between March 2020 and August 2020. The data for services were collected based on the harmonized definitions as per the DESDE coding system for PECUNIA to ensure that the costing information was collected for services with comparable scope in all PECUNIA partner countries. The unfortunate concurrence with the first wave of the COVID-19 pandemic in Austria substantially curbed the possibilities for the collection of primary data for service-type resource use items and the initially foreseen primary data collection directly from service providers was ultimately not feasible in many cases. In these instances, secondary data were used instead. The PECUNIA RUC Templates and their accompanying user guide provide detailed suggestions for data collection (e.g., the minimum number of service providers that should be contacted, or when and how secondary data can be used instead). The data were extracted from diverse sources. Table 3 summarizes the key details of the data types and data source for each resource use item.

Data on healthcare services were obtained from the Main Association of Austrian Social Security Institutions and based on routine data, with the exception of the RUC for health-related support line, for which data were taken from a publicly available annual report from a main nationwide service provider. Similarly, data on social services (nursing home, day care centers) were obtained through public and non-public reports from the National Statistics Office (Statistics Austria), data on educational services were taken from publicly available research reports, and employment-related productivity figures (absenteeism/presenteeism, friction period) were extracted from publicly available reports by the Public Employment Service.

We used proxy measures for the RUCs for police contacts and contacts with non-contract general practitioners (GPs) and dentists. While for police contacts we based our RUC estimate on the official fee charged when police officers provide security for mass events, we consulted (non-binding) fee recommendations for non-contract GP and dentist contacts. Lastly, primary data were used for the RUC estimates for unpaid labor and informal care, for which we used the prices of 10 service providers each based on an online search.

### PECUNIA RUC calculation

The data collected for the different resource use items were in the next step entered in the corresponding resource use-specific PECUNIA RUC Templates [37]. In cases when the data collection already yielded the desired figure, these were used directly as PECUNIA RUCs after ensuring that all relevant cost components were included and that the estimates were in line with the PECUNIA costing methods. For services, three different PECUNIA RUC Templates have been developed and tailored to granularity of the available data: the SERVICE‑1 Template (top-down micro-costing), the SERVICE‑2 Template (top-down gross-costing), and the truncated SERVICE‑2 Short Template (top-down gross-costing), which aims to minimize the burden of data collection. The PECUNIA RUC Templates further include dedicated templates for non-service costs related to ‘TANGIBLE CONSEQUENCES’ (e.g. vandalism), ‘PRODUCTIVITY LOSS’ (e.g. absenteeism) and ‘PERSONAL TIME’ (e.g. informal care). Table 1 highlights which PECUNIA RUC Template were used in the calculation for each final PECUNIA RUC for Austria. Additionally, the PECUNIA costing tools include the PECUNIA RUC Aggregation/Weighting (RAW) data sheets that enable researchers to develop an aggregated estimate based on multiple unit cost estimates for the same resource use item (e.g. from multiple data or service providers).

**Table 1:** Data type, data source and PECUNIA Reference Unit Cost (RUC) Templates used for the 22 PECUNIA RUC estimates for Austria in health and social care, education, (criminal) justice, employment, and informal care

No.	Resource (use) item (National language name)	Data type	PECUNIA Reference Unit Cost (RUC) Template used	Data source
01	Dental care (Zahnbehandlung)	Secondary data	SERVICE‑2 short	Statistical Handbook of the Austrian Social Insurance Funds [43]; additional information on contacts per case received from Main Association of Austrian Social Security Institutions
02	Dental care (Zahnbehandlung)	Secondary data	Not used	Autonomous fee guidelines of the Austrian chamber of dentists [44] 2019/2020 according to § 19 (2) Zeile 5 Zahnärztekammergesetz (ZÄKG), per session (per hour): 60 min, assumption: average consultation with a ‘private’ dentist = 30 min
03	Dental care (Zahnbehandlung)	Secondary data	SERVICE‑2 short	Weighted average of unit cost estimates No. 01 and No. 02; weights: medical ambulatory care unit (Ärztlich ambulante Versorgungseinheit, ÄAVE, 2018). Weights based on Regiomed data provided by the Main Association of Austrian Social Security Institutions
04	General practitioner (Allgemeinmedizinerkonsultation)	Secondary data	SERVICE‑1	Physicians’ cost statistics (Ärztekostenstatistik) 2015; share of overhead costs: Waldner (2001) [45]; direct-indirect time ratio and average length of consultation: Hoffmann et al. (2015) [46]
05	General practitioner (Allgemeinmedizinerkonsultation)	Secondary data	Other (see additional comments)	(Non-binding) price recommendations by the regional physicians’ chambers for non-contracted physicians from six federal states, average, for source see Mayer S et al. 2020 [47]
06	General practitioner (Allgemeinmedizinerkonsultation)	Secondary data	SERVICE‑1	Weighted average of unit cost estimates No. 04 and No. 05; weights: medical ambulatory care unit (Ärztlich ambulante Versorgungseinheit, ÄAVE, 2018). Weights based on Regiomed data provided by the Main Association of Social Security Institutions
07	Health-related day care centre (Teilstationäre Tagesbetreuung)	Secondary data	SERVICE‑2 short	Statistik Austria, Care statitics (Pflegedienstleistungsstatistik) 2018; [48]
08	Health-related support line, mental health (Notruf-Hotline für Kinder und Jugendliche – Rat auf Draht)	Secondary data	SERVICE‑2 short	“Rat auf Draht” annual report 2019 [49]; for average length of phone contact: Rat auf Draht Jahresbericht 2018 [50]
09	Nursing home (Stationäre Betreuungs- und Pflegedienste)	Secondary data	SERVICE‑2 short	Bayerl N (2020) [51]
10	Education services provided in a special education school (Sonderschule)	Secondary data	SERVICE‑2 short	National education report Austria (Nationaler Bildungsbericht Österreich) 2018 [52], email communication with an author of the report
11	Educational therapy – primary school (Förderunterricht – Primärstufe)	Secondary data	SERVICE‑2 short	Austrian Court of Audit, General Report on Income (Rechnungshof Allgemeiner Einkommensbericht) 2018 [53]; email communication expert; relevant minimum number of students taken from the relevant law (Bundesrecht konsolidiert: Gesamte Rechtsvorschrift für Eröffnungs- und Teilungszahlenverordnung, Fassung vom 14.04.2014)
12	Educational therapy – secondary school (Förderunterricht – Sekundarstufe)	Secondary data	SERVICE‑2 short	Austrian Court of Audit, General Report on Income (Rechnungshof Allgemeiner Einkommensbericht) 2018 [53]; email communication with expert; relevant minimum number of students taken from the relevant law (Bundesrecht konsolidiert: Gesamte Rechtsvorschrift für Eröffnungs- und Teilungszahlenverordnung, Fassung vom 14.04.2014)
13	Incarceration in jail (Gefängnisaufenthalt)	Unknown	Not used	Federal Ministry of Justice (Bundesministerium für Justiz) (2016) [54]: Strafvollzug in Österreich, p. 41
14	Police contact (Polizeikontakt)	Unknown	Not used	Federal law, consolidated: Gesamte Rechtsvorschrift für Sicherheitsgebühren-Verordnung 2022 [55]
15	Police contact (Polizeikontakt)	Unknown	Not used	Federal law, consolidated: Gesamte Rechtsvorschrift für Sicherheitsgebühren-Verordnung 2022 [55]
16	Property damage – Car (Sachbeschädigung – Auto)	Secondary data	TANGIBLE CONSEQUENCES	Austrian Insurance Association, Annual Report (Österreichischer Versicherungsverband, Jahresbericht) 2018 [56], damage and benefit cases, p. 105 and p. 136
17	Absenteeism/presenteeism (Absentismus/Präsentismus)	Secondary data	Not used	Statistik Austria, labor cost statistics (Arbeitskostenstatistik) [57]
18	Friction period (Friktionsperiode)	Secondary data	PRODUCTIVITY LOSS	Public Employment Service (Arbeitsmarktservice Österreich): Arbeitsmarktlage 2019 [58]
19	Unpaid work (Unbezahlte Arbeit)	Secondary data	PRODUCTIVITY LOSS	Statistik Austria, Personal income, 2020 [59]
20	Unpaid work (Unbezahlte Arbeit)	Primary data	PRODUCTIVITY LOSS	Search on online platform to find household help: 2020 Haushaltshilfe24 [60]
21	Retirement age (Pensionsantrittsalter)	Unknown	Not used	Information by the Pension Insurance Institution, 2020 [61]
22	Informal care (Informelle Pflege)	Primary data	PERSONAL TIME	Online search for the prices from 10 regional providers; access date 24 April 2020; links to the providers available upon request

The cost estimate for a given resource use item may further depend on the funding sources. Therefore, we have calculated RUC estimates separately for (i) state/social insurance funded items, (ii) privately funded items, or (iii) as a (weighted) representative mixed estimate where necessary. This is relevant in countries with parallel systems of publicly and privately paid healthcare segments, as is the case in Austria. The category (iv) ‘other’ indicates funding through alternative channels, e.g., via donations. For this reason, the PECUNIA RAW datasheets also include an optional weighting of the individual unit cost estimates in the RUC based on market share or population size. In the present set of RUCs for Austria, this weighting option was used for the estimates for GP and dentist contacts, which accounts for visits to GPs with and without a contract with an Austrian sickness fund.

### External validation

The concluding step of the PECUNIA RUC development was a structured external validation of the preliminary RUC estimates calculated by the PECUNIA researchers. In general, this step allows researchers who are developing RUCs to assess critically the validity of the RUC estimate through comparison against existing unit costs or with feedback from experts or data providers. The external valuation proved to be a valuable step in the PECUNIA project, in particular when using secondary data. For instance, when we reached out to the Statistics Austria (the data provider) for the validation of the preliminary estimate for nursing homes, the National Statistics Office provided us with non-public, novel data using national accounts that captured the cost structure of nursing homes more accurately compared to the other publicly available data [51].

We further combined the outcome of the external validation with an indicator for compliance with the PECUNIA costing standards (where full compliance implies that RUCs are representative at the national level, are based on a top-down gross-costing or micro-costing approach, and with no major data limitations being reported) to an overall level of certainty quality indicator reported for each PECUNIA RUC. The combined index provides future users of the PECUNIA RUC Compendium with an intuitive indicator for the reliability of each RUC estimate and draws attention to any issues that need to be taken into consideration when using a specific RUC.

## Results

In the course of the PECUNIA project, we calculated 22 RUCs for the reference year 2019 for Austria, covering the sectors health and social care, education, (criminal) justice, employment, and patient, family and informal care. We discuss the Austrian RUCs separately for each sector. Table 2 presents the summary statistics of the set of 22 RUCs for Austria, while Table 3 provides the RUC figures and details. We report the Austrian PECUNIA RUCs in Euros (EUR) adjusted to the price level of the reference year 2019. The non-monetary RUCs (friction period and retirement age) are expressed in calendar days and years, respectively.

**Table 2:** Summary statistics on the main characteristics of the 22 PECUNIA Reference Unit Cost (RUC) estimates for Austria in the health and social care, education, (criminal) justice, employment, and informal care

Reference Unit Cost (RUC) characteristics (*N* = 22)	*n* (%)
*Unit cost type*	
PECUNIA RUC	16 (73)
Existing unit cost estimate	6 (27)
*Data type*	
Primary data	2 (9)
Secondary data	16 (73)
Combined data	–
Unknown/undisclosed	4 (18)
*Original year(s) of RUC*	
2015 or older	3 (14)
2016	2 (9)
2017	3 (14)
2018	5 (23)
2019	4 (18)
2020	4 (18)
Multiple years	1 (5)
*External validation process*^a^	
Comparison to existing unit cost estimate	8 (36)
Expert feedback	10 (45)
Data provider feedback	7 (32)
No external evaluation	8 (36)
*Direct match with PECUNIA Ressource Use Measurement (RUM) items*	
Matching	18 (82)
Not matching	4 (18)

^a^Note that some RUCs have been externally validated using more than one approach

**Table 3:** Summary of the 22 PECUNIA Reference Unit Cost (RUC) estimates for Austria in health and social care, education, (criminal) justice3, employment, and informal care (in EUR, for year 2019)

No	Resource (use) item (National language name)	Funding source	Unit of measurement	DESDE code	Reference Unit Cost^a^	Representativeness of RUC	Direct match with PECUNIA RUM instrument	Unit cost calculation approach	Overall certainty of RUC
01	Dental care (Zahnbehandlung)	State/social insurance-funded	Per contact	SH-NX [K00-K14] O8.1	€94.99	National	✘	Top-down gross-costing	2 (medium)
02	Dental care (Zahnbehandlung)	Privately funded	Per contact	SH-NX [K00-K14] O8.1	€120.00	National	✘	Unknown	2 (medium)
03	Dental care (Zahnbehandlung)	Representative average	Per contact	SH-NX [K00-K14] O8.1	€96.92	National	✔	Composite	2 (medium)
04	General practitioner (Allgemeinmedizinerkonsultation)	State/social insurance-funded	Per contact	SH-NX [ICD-10] O8.1	€31.80	National	✘	Top-down micro-costing	1 (high)
05	General practitioner (Allgemeinmedizinerkonsultation)	Privately funded	Per contact	SH-NX [ICD-10] O8.1	€45.45	National	✘	Unknown	2 (medium)
06	General practitioner (Allgemeinmedizinerkonsultation)	Representative average	Per contact	SH-NX [ICD-10] O8.1	€32.04	National	✔	Composite	1 (high)
07	Health-related day care center (Teilstationäre Tagesbetreuung)	State/Social insurance-funded – applicable only	Per day	SH-NX [ICD-10] D4.1; SH-NX [F00-F99] D4.1; SS-NX [ICF] D4.1	€78.93	National	✔	Top-down gross-costing	2 (medium)
08	Health-related support line, mental health (Notruf-Hotline für Kinder und Jugendliche – Rat auf Draht)	Other	Per contact	SH-NX [F00-F99] I1.2.4e	€9.98	National	✔	Top-down gross-costing	1 (high)
09	Nursing home (Stationäre Betreuungs- und Pflegedienste)	Representative average	Per night	SS-OX-R11; SH-AO [F00-F99] R11; SH-AO [ICF] R11	€177.82	National	✔	Top-down gross-costing	1 (high)
10	Education services provided in a special education school (Sonderschule)	State/social insurance-funded	School day	SE-CX [F7-F9][ICF] D4.2g	€188.85	National	✔	Top-down gross-costing	1 (high)
11	Educational therapy – primary school (Förderunterricht – Primärstufe)	State/Social insurance-funded	Per contact	SE-CC [F7-F9] - O8.2	€7.73	National	✔	Top-down gross-costing	3 (low)
12	Educational therapy – secondary school (Förderunterricht – Sekundarstufe)	State/Social insurance-funded	Per contact	SE-CA [F7-F9] - O8.3	€5.82	National	✔	Top-down gross-costing	3 (low)
13	Incarceration in jail (Gefängnisaufenthalt)	State/Social insurance-funded	Per night per person	SJ-AO [Z65.1] R8.2ci	€130.07	National	✔	Top-down gross-costing	2 (medium)
14	Police contact (Polizeikontakt)	State/Social insurance-funded	Per contact (30 minutes)	SJ-NX [F0-F9]-O1.2	€47.00	National	✔	Unknown	3 (low)
15	Police contact (Polizeikontakt)	State/Social insurance-funded	Per contact (1 hour)	SJ-NX [F0-F9]-O1.2	€94.00	National	✔	Unknown	3 (low)
16	Property damage – Car (Sachbeschädigung – Auto)	Not applicable	Per single incident	Not applicable	€1772.93	National	✔	Top-down gross-costing	3 (low)
17	Absenteeism/presenteeism (Absentismus/Präsentismus)	Not applicable	Per hour	Not applicable	**€**34.35	National	✔	Not applicable	1 (high)
18	Friction period (Friktionsperiode)	Not applicable	Calendar days	Not applicable	89 days	National	✔	Not applicable	1 (high)
19	Unpaid work (Unbezahlte Arbeit)	Not applicable	Per hour	Not applicable	**€**13.50	National	✔	Not applicable	1 (high)
20	Unpaid work (Unbezahlte Arbeit)	Not applicable	Per hour	Not applicable	**€**11.11	Local	✔	Not applicable	2 (medium)
21	Retirement age (Pensionsantrittsalter)	Not applicable	Years of age	Not applicable	63.5 years	National	✔	Not applicable	1 (high)
22	Informal care (Informelle Pflege)	Other	Per hour	Not applicable	€14.07	National	✔	Not applicable	1 (high)

^a^Monetary values are adjusted to 2019 price levels

Currently, 18 of the 22 RUCs are directly matched with items in the PECUNIA Resource Use Measurement (RUM) instrument, which collects information on resource use in more general terms and relies on the representative average RUC estimate in case of multiple possible funding sources.

### Health and social care services

We calculated nine PECUNIA RUCs for five resource use items in the health and social care sectors for Austria. For GP consultations and dental care services, three RUCs were calculated each, to reflect differences in the cost depending on the funding source. For both services, healthcare service contacts occur by and large within the framework of the Austrian social health insurance system with its network of contract physicians and near-universal coverage. Only a small fraction of contacts happens outside it, i.e., with patients covering all expenses for a physician visit out-of-pocket, either because patients visit non-contract physicians, or very rarely, are not insured with an Austrian sickness fund. Hence, the representative average estimates weighted by the number of contacts in each setting of €32.04 per GP contact and €96.92 per dental care contact are much closer to the RUCs calculated for social health insurance-funded contacts (€31.80 per GP contact; €94.99 per dental care contact) than to the RUCs calculated for privately funded contacts (€45.45 per GP contact; €120.00 per dental care contact). Data limitations reduce the certainty of the RUCs for dental care and privately funded GP contacts to medium, but both RUCs have been positively externally validated. The RUC estimates for mental health-related support lines of €9.98 per contact and nursing homes stays of €177.82 per night—derived from national accounts for greater precision [51]—are of high quality, while the expert feedback on the RUC for health-related day care centers of €78.93 per day cautioned that the costs of providing this service per client vary strongly depending on the size of the provider, resulting in a medium level of certainty overall.

### Education

The set of PECUNIA RUC estimates includes three RUC estimates for Austria for three resource use items in the education sector. We report two separate RUCs of the resource use item “educational therapy” for primary and secondary schools due to the difference in the minimum/maximum class sizes mandated by federal law. While in secondary schools, the minimum (maximum) size of 8 (12) students in educational therapy classes results in an RUC estimate of €5.82 per student contact, the lower minimum (maximum) size of 3 (8) students in primary schools for educational therapy classes results in slightly higher RUC estimate of €7.73 per student contact. The level of certainty of both RUC estimates is marked as low due to the absence of external validation, and both estimates are based solely on staff cost for teachers, thereby omitting important cost categories, for instance, overhead costs from administration, etc. In contrast, for education services provided in special education schools, no distinction between primary and secondary education is necessary. The resulting RUC estimate of €188.85 is of high certainty, as it covers all relevant direct and overhead costs, and has received positive expert validation.

### (Criminal) justice

In the (criminal) justice sector, we calculated four RUCs for three resource use items in Austria. Notably, we report two RUCs for police contacts of €47 and €94 per half-hour to 1h contacts, respectively, based on the duration of the police contact. As we derived the RUCs from a legal fee charged for police officers providing security for mass events, the quality of the estimate is low. For property damage to cars through vandalism, we estimate an RUC at €1772.93 per single incident, but due to limitations in the data, which include all types of damages to cars (e.g., traffic accidents) and not just vandalism, the certainty of the RUC is low. Lastly, we estimated the RUC of incarceration in jail per night at €130.07. The level of certainty is medium, as the estimate is based on full costs, but no external validation is available.

### Employment and productivity

We calculated a total of five RUCs for four resource use items for the employment and productivity sector. The RUC for absenteeism/presenteeism of €34.35 per hour was based on an official statistic of the average labor costs per hour actually worked and hence of high quality. In a similar way, the RUC estimate for the national retirement age of 63.5 years was based on the official retirement age as intended by the lawmaker (mean between female and male legal retirement age). The estimated RUC for the friction period of 89 calendar days was based on the filled and unfilled vacancies plus 28 days of training. The certainty of the estimate is high as it compares favorably with the officially reported statistics for Austria [58]. Lastly, for unpaid work, two RUC estimates are available, both capturing the hourly replacement costs for home help but varying by the type of data used. The first estimate of €13.50 builds on secondary data and corresponds to the average hourly gross wage for care workers. The certainty of this estimate is reported as high as it is valid at the national level and compares favorably to the results of a recent study on the value of household work in Austria [62]. In contrast, the second RUC estimate of €11.11 is based on primary data and—similar to the method recently used by Jokubauskaitė and Schneebaum [62] for a comparable task—imputes the value of housework from the actual local wage rate as requested for such services on an online platform. As this RUC estimate is only valid at the local level, it is only assigned medium certainty.

### Patient and family domains – informal care

For the domains of patient and family, we report 1 RUC estimate for informal care based on the mean of the prices from 10 regional providers. The RUC estimate of €14.07 compares favorably with estimates for the hourly national minimum and average wages and is hence endowed with high certainty. However, the arguably large span between the minimum (€3.37) and maximum (€32.10) prices used for the RUC estimation suggests that the costs vary strongly between providers depending on the federal state and whether the provider is a private or public enterprise.

## Discussion

In this article, we present the 22 cross-sectoral PECUNIA RUC estimates developed for Austria that researchers, policy makers, and professionals can use in health economic evaluations of policies or interventions, or any other type of cost analysis that benefits from the inclusion of a societal perspective. The cross-country comparable RUC estimates are so far available online for five other countries (Germany, Hungary, the Netherlands, England/UK, and Spain) in the multi-sector, multi-country PECUNIA RUC compendium [27], which is available free of charge for non-commercial purposes (https://pecunia-project.eu/tools/ruc-compendium). In combination with the other PECUNIA costing tools—PECUNIA Templates for costing (https://pecunia-project.eu/tools/ruc-templates) and the PECUNIA RUM instrument for resource use measurement (https://pecunia-project.eu/tools/rum-instrument), the PECUNIA RUCs offer the possibility to make it easier to adopt a comprehensive societal perspective in health economic evaluations due to the improved cross-sectoral coherence of methods and estimates. Apart from the cross-sectoral validity, the improved cross-country comparability of the PECUNIA RUCs further opens up new possibilities for health economic evaluations in an international context. Interested readers can find a detailed discussion of the cross-country comparability of the PECUNIA RUCs for health and social care services in a recent publication by Mayer et al. [26]. In the context of Austria, this is the first set of specifically calculated unit costs based on a harmonized methodology, thereby contributing to the data pool for future research to ensure a high-quality evidence base for healthcare decision making. The PECUNIA RUCs are in general not adjusted for purchasing power parity (PPP), as national level unit costs are typically the starting point even of multinational economic evaluations. Hence, the PECUNIA RUCs for Austria reported in this article are ready to use in economic evaluations in the Austrian healthcare system and do not need additional adjustment. Note that the information reported in Table 3 provides only a selection of RUC characteristics reported in the PECUNIA RUC compendium. The full PECUNIA RUC compendium includes more detailed information such as the ranges of the RUC estimates (minimum and maximum values of provider-specific or region-specific unit costs) or further information on weighting (e.g., staff grade). These metadata are transparently documented in the PECUNIA costing tools to allow for further updating of given RUCs and are also explained in detail in complementing user guides.

The PECUNIA RUCs also follow clear methodological guidelines that reduce the impact of unaccounted biases in the estimates by explicitly specifying first-best options to avoid the typical pitfalls of using non-optimal proxies for cost estimates. For instance, using tariffs, which usually contain (substantial) profits, in value-based pricing would disproportionally shift welfare gains created by novel technologies from patients to producers [63]. Moreover, the possibility to transparently differentiate RUCs according to the funding source has the advantage that such granulated RUCs allow for a more precise estimate of the economic impacts along socioeconomic strata. For instance, when policy makers decide to roll back the publicly funded provision of a healthcare service to alleviate pressure from public budgets, patients would have to substitute their healthcare needs with privately funded services. This implies two important considerations: first, privately funded services are for several reasons (usually bargaining power and amenities, but occasionally also service quality) often more cost-intensive than publicly funded healthcare services, hence the public savings represented by the lower unit cost estimate of publicly funded services would be disproportionally offset by higher expenditures in the private domain. At the same time, less affluent patients might either not be willing or able to make this substitution [64]. The granulated funding source-specific RUCs allow for a much more accurate cost assessment and facilitate finely tuned policy making to avoid unintended adverse effects by limiting access to care for less affluent patients.

Comparing the PECUNIA RUCs to the previously available unit costs estimates for healthcare services in Austria in the DHE Unit Cost Database [22], the key advantage of the precise and unambiguous resource use definitions becomes clear. This can be illustrated on the example of the seemingly straightforward example of GP visits. As the DHE Unit Cost Database is based on a systematic screening of the available literature with the purpose of providing a complete picture of available estimates, it includes several estimates for this item that may use different wording (e.g., GP visit or physician visit). Some unit estimates were further derived for very specific disorders (e.g. contacts related to dementia in contrast to chronic heart failure) and may relate to very different units of measurement (e.g., per minute or per contact) or years. Unsurprisingly, the unit cost estimates for this common resource use item cover a wide range from €10 to €40. For other resource items for which comparable unit cost estimates are also available in the database (productivity loss and informal care), similar problems are observed. Other unit cost estimates (nursing homes and home help as a proxy for unpaid labor) are only based on data from one provider and might hence be prone to provider-specific bias.

The improved quality of the evidence base for health economic decision making will facilitate bringing Austrian Health-in-All-Policies activities in line with their intended goals through greater precision in the impact assessment of economic shocks and the optimization of cross-sectoral funding arrangements. This creates new maneuvring space in terms of financial sustainability of healthcare systems. These fiscal challenges are manifold and not restricted to Austria alone. For one, many countries have observed a widening rift between economic growth and the growth of healthcare expenditure due to a complex interplay of demographic, social, economic, technological and institutional factors [65]. Adopting a societal perspective in economic evaluations will allow a more accurate capture of the spill-over costs and benefits between sectors, thereby accounting for the intricate intersectoral connections. Additionally, we know from recent negative economic shocks (e.g., the Great Recession of 2007–2009) that the decision on which screws to turn when trying to realign budgets and healthcare spending is largely a political choice, albeit often contingent both on the general economic climate as well as the economic agenda of large international lender institutions like the International Monetary Fund (IMF) [66]. In the aftermath of the economic disruption caused by the COVID-19 pandemic through 2020 and 2021 and the global economic turmoil through the unfolding Ukrainian crisis in 2022, the issue of managing healthcare budgets will likely move up the political agenda again in various forms. For instance, government debt in Austria has already steeply increased from 70.5% of the GDP in 2019 to 83.9% of the GDP in 2021 [67]. In this respect, efficiency gains in the healthcare system and better prioritization could prevent adverse public health consequences of austerity measures (see, e.g., [68]) in case political decision makers in Austria or elsewhere are inclined to move in this direction again. The horizon of this discussion extends beyond the immediate budgetary impact of economic crises. Novel technologies in pharmaceuticals, for instance in cancer care, can provide substantial gains in survival, but the attached price tag can pose a challenge. Costs of USD140,000–150,000 per quality-adjusted life year gained [69] are not unusual. Even in times when public budgets are not being squeezed by economic crises, such numbers are challenging for decision makers. Again, Austria is not alone in facing this issue, which concerns decision makers not just in Europe but in fact around the globe. The inclusion of a societal perspective in cost-efficiency considerations of drug reimbursement or in the process of health technology assessment (HTA) in general, may prove particularly helpful as it allows a more complete capture of the medium-term to long-term benefits, such as when patients are not lost to the workforce, etc.

Lastly, in the context of future endeavours in developing new unit cost estimates, we consider two practical aspects of the PECUNIA tools to be key assets. While the PECUNIA tools have the potential to substantially reduce the time required for the calculation of harmonized unit costs, the inclusion of an external validation in the workflow improves the validity of PECUNIA RUCs as demonstrated by the PECUNIA RUC for nursing home stays that has an edge in precision over common alternative sources like public expenditures on nursing home services. Based on our experience in the PECUNIA project, we strongly encourage researchers to include external validation processes in future unit cost development, notwithstanding whether researchers use the PECUNIA tools and methods or not, because of its potential to improve data quality.
